# Severe cutaneous adverse reaction to aumolertinib plus vebreltinib in an EGFR-mutant NSCLC patient: a case report

**DOI:** 10.3389/fonc.2026.1935204

**Published:** 2026-10-28

**Authors:** Yaoyao Zhang, Luxin Zhang, Jiyuan Ding

**Affiliations:** 1 Department of Oncology and Hematology, Hangzhou Red Cross Hospital, Hangzhou, China; 2 Second Clinical Medical College, Zhejiang Chinese Medical University, Hangzhou, China

**Keywords:** aumolertinib, case report, non-small cell lung cancer, Stevens-Johnson syndrome, toxic epidermal necrolysis, vebreltinib

## Abstract

**Background:**

The combination of aumolertinib and vebreltinib has emerged as an effective therapeutic strategy for advanced non-small cell lung cancer(NSCLC) harboring EGFR mutations with concurrent MET amplification. However, the safety profile of this combination regimen remains incompletely characterized, particularly with respect to severe cutaneous adverse reactions.

**Case presentation:**

We report a 72-year-old female patient with NSCLC harboring EGFR 19Del and MET amplification. Fifteen days after initiating treatment with the combination of aumolertinib and vebreltinib, the patient developed severe oral mucositis and rapidly progressive generalized exfoliative dermatitis, clinically suspected as Stevens-Johnson syndrome/toxic epidermal necrolysis (SJS/TEN) or an SJS/TEN-like reaction. No skin biopsy was performed because the family declined; therefore, histopathological confirmation was unavailable. The patient was admitted on February 5, 2026. SCORTEN was calculated using the available admission parameters and was 4. Initial treatment with intravenous methylprednisolone (40mg QD) for three days was of limited efficacy; therefore, the dose was escalated to 60mg BID, and intravenous immunoglobulin (IVIG) and human albumin were added. Due to inadequate response after four days of high-dose corticosteroid therapy, a single dose of the TNF-α inhibitor adalimumab (80mg) was administered subcutaneously as a rescue therapy. The patient was transferred to a specialized dermatology hospital the following day. The patient was transferred to a specialized dermatology hospital the following day. Telephone follow-up indicated clinical improvement after continued adalimumab plus corticosteroids; she was discharged after four weeks. However, complete external records and discharge photographs were unavailable, and the effectiveness of adalimumab cannot be established from this case.

**Conclusion:**

The combination of aumolertinib and vebreltinib has been associated with a rare but life-threatening suspected SJS/TEN-like reaction that can be refractory to conventional-dose corticosteroids. Adalimumab plus corticosteroids was administered as rescue therapy in this case, but its efficacy cannot be determined based on a single case and incomplete follow-up. Clinicians should maintain a high index of suspicion for this cutaneous toxicity, promptly discontinue suspected drugs, and arrange specialized care.

## Introduction

Epidermal growth factor receptor tyrosine kinase inhibitors (EGFR-TKIs) are the cornerstone treatment for EGFR-mutated advanced NSCLC. However, acquired MET amplification is a prevalent mechanism of resistance to first-line EGFR-TKI therapy, and combination regimens targeting this mechanism have emerged as critical therapeutic strategies (1). The combination of aumolertinib, a third-generation EGFR-TKI, and vebreltinib, a highly selective MET inhibitor, has emerged as a promising approach in clinical practice (2, 3).

Although rash and stomatitis are common class-effect adverse reactions associated with EGFR-TKIs, life-threatening severe cutaneous adverse reactions, following combination targeted therapy remain exceedingly uncommon (4).Stevens-Johnson syndrome/toxic epidermal necrolysis(SJS/TEN) represents a spectrum of severe mucocutaneous hypersensitivity reactions characterized by widespread epidermal detachment, mucosal involvement, and high mortality rates, with an incidence of approximately 1–12 per million person-years for medication-related cases (5).

Herein, we report a case of suspected SJS/TEN-like reaction following aumolertinib combined with vebreltinib. We also describe the clinical characteristics, discuss potential management strategies, and seek to enhance clinical awareness of this severe adverse event. As no skin biopsy was obtained, the diagnosis remains suspected throughout the manuscript.

## Case report

### Patient information and medical history

A 72-year-old female patient (height: 160 cm; weight: 51kg) was admitted on February 5, 2026, presenting with a one-day history of skin rash and oral ulcers following targeted therapy for lung malignancy diagnosed two years earlier. On admission, the vital signs were: body temperature 37.1 °C, heart rate 102 beats/min, oxygen saturation 96%, and a blood pressure of 122/91 mmHg.

Comorbidities: Patient denies any history of hypertension, diabetes, chronic liver disease, chronic kidney disease, or other chronic conditions.

Concomitant medications before admission: The patient reports no use of any other medications.

Allergy history: The patient reports no history of drug allergies.

Laboratory findings at admission: As shown in **Table 1**.

**Table 1:** Admission laboratory findings.

Variable	Reference range	Result
Complete blood count		
WBC (×10^9^/L)	3.5–9.5	3.3 ↓
Neutrophils (×10^9^/L)	1.8–6.4	2.97
Lymphocytes (×10^9^/L)	1.0–3.3	0.26 ↓
Eosinophils (×10^9^/L)	0–0.3	0.04
Basophils (×10^9^/L)	0–0.15	0
Hemoglobin (g/L)	110–150	120
Platelets (×10^9^/L)	100–300	43 ↓
Biochemical profile		
Glucose (mmol/L)	3.9–6.1	9.0 ↑
ALT (U/L)	7–40	86 ↑
AST (U/L)	13–35	133 ↑
Total bilirubin (μmol/L)	3.4–20.5	3.84
Creatinine (μmol/L)	41–111	112 ↑
BUN (mmol/L)	3.1–8.8	13.3 ↑
Albumin (g/L)	40–55	29.8 ↓
Sodium (mmol/L)	137–147	135 ↓
Potassium (mmol/L)	3.5–5.3	3.65
Total calcium (mmol/L)	2.15–2.57	1.89 ↓
hs-CRP (mg/L)	0–3.3	136.19 ↑
PCT (ng/mL)	0–0.05	1.25 ↑
Coagulation profile		
PT (s)	10.5–14.3	10.8
APTT (s)	18–34	40.5 ↑
D-dimer (μg/L)	0–750	6072 ↑
Fibrinogen (mg/dL)	170–500	217

The patient underwent a lung mass biopsy two years prior, with pathological results indicating non-small cell lung cancer, which was consistent with adenocarcinoma based on immunohistochemistry. Immunohistochemical findings were as follows: CK5/6 (weakly positive), P53 (-), P63 (-), TTF-1 (+), P40 (-), Ki-67 (+7%), Napsin A (+), CK7 (+), INSM1 (-), PD-1 (<1%). The diagnosis was pulmonary adenocarcinoma with adrenal metastases, and the patient had an Eastern Cooperative Oncology Group (ECOG) performance status of 1-2. Preliminary genetic testing revealed a deletion in exon 19 of the EGFR gene. Initial treatment involved continuous first-line osimertinib therapy for six months; follow-up imaging demonstrated progression of the pulmonary lesion, suggesting acquired resistance. Subsequent bronchoscopy with biopsy and repeat genetic testing confirmed the persistence of the original EGFR exon 19 deletion and identified MET gene copy number amplification as the mechanism of osimertinib resistance.

### Treatment and adverse reaction onset

Based on the identified resistance mechanism, the treatment regimen was modified to a combination of aumolertinib (110mg once daily) and vebreltinib (300mg once daily).

On day 15 of combination targeted therapy (one day prior to admission), the patient developed severe oral ulcers, causing intense pain and preventing oral intake. Concurrently, diffuse erythema appeared on the trunk, back, and face, which rapidly progressed to extensive bullae formation and exfoliative dermatitis (**Figure 1**). The Nikolsky sign was positive. Involvement of the ocular or genital mucosa was not observed. The involved body surface area exceeded 30%, and the patient was clinically suspected of having SJS/TEN overlap or TEN.

**Figure 1:**
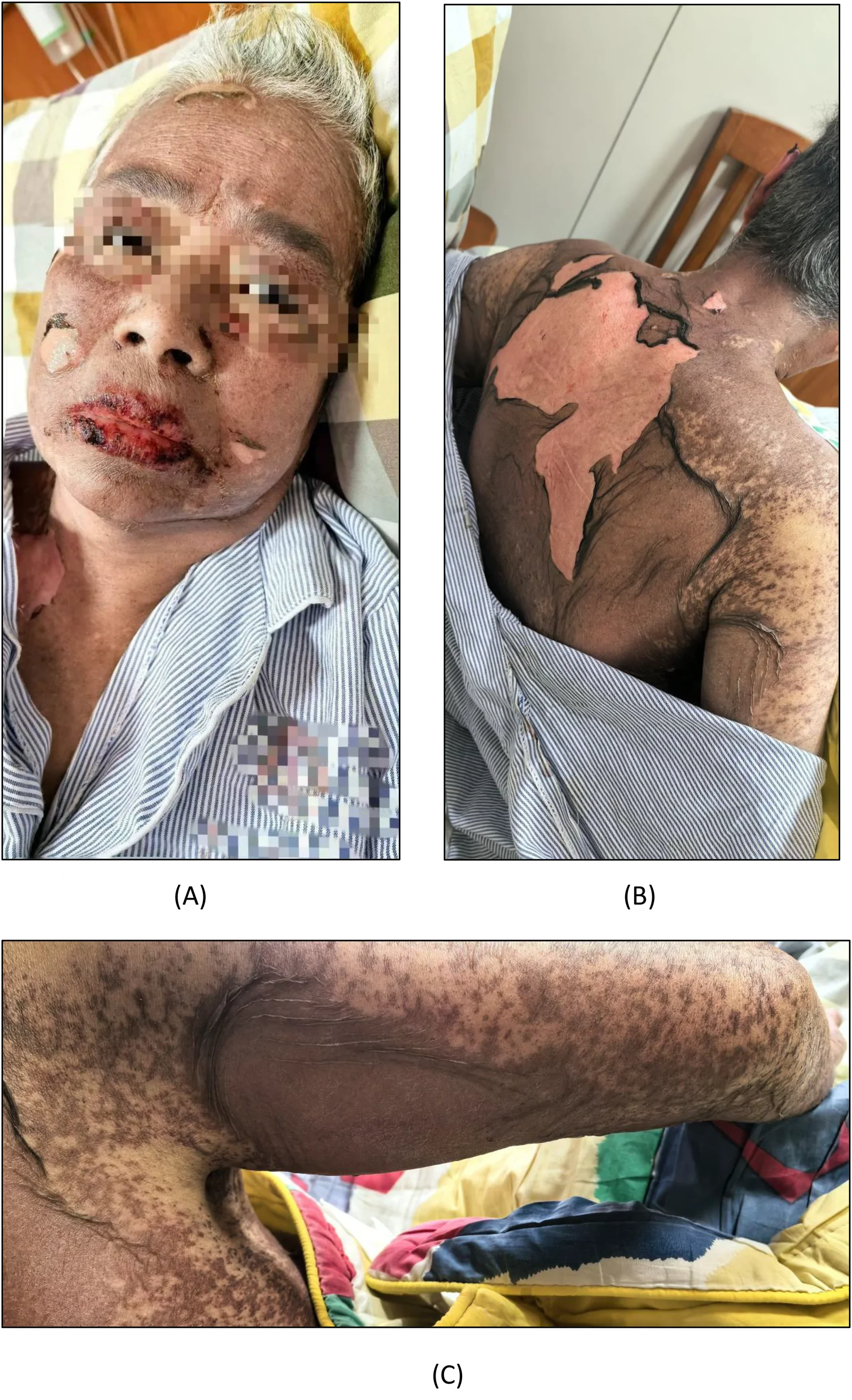
Clinical manifestations on admission. **(A)** Facial and neck involvement with severe oral mucositis and hemorrhagic crusting. **(B)** Trunk involvement demonstrating extensive erythema and bullae. **(C)** Limb involvement showing widespread skin detachment.

### Differential diagnosis

Given that no skin biopsy was performed, the differential diagnosis included erythema multiforme major, generalized bullous fixed drug eruption, acute generalized exanthematous pustulosis (AGEP), drug reaction with eosinophilia and systemic symptoms (DRESS), and other drug-related exanthems. Erythema multiforme major typically presents with target lesions and less extensive epidermal detachment; generalized bullous fixed drug eruption typically presents with a history of exposure to the same drug and more localized lesions; AGEP typically presents with pustules and neutrophilia; DRESS typically presents with eosinophilia and organ involvement. In this patient, the combination of mucosal erosions, a positive Nikolsky sign, body surface area involvement of >30%, and rapid progression supported a suspected SJS/TEN-like reaction. However, histopathological confirmation would be required for a definitive diagnosis and was unavailable.

### Hospital course and treatment

February 5, 2026 (Hospital Day 1):Upon admission, all targeted therapies were promptly discontinued. A skin biopsy was strongly recommended to confirm the diagnosis; however, the family declined the procedure. Given high clinical suspicion of SJS/TEN-like reaction, supportive therapy was initiated, including oral care with vitamin B complex and lidocaine mouthwash. Because of the elevated risk of secondary infection in exposed skin areas, empirical sulbactam–ampicillin (3g every 8 hours) plus vancomycin (1g every 12 hours) was administered. Intravenous methylprednisolone (40mg once daily) was started. SCORTEN was calculated using available admission parameters: age, malignancy, body surface area, urea, bicarbonate, glucose, and heart rate; total score 4, predicted mortality 58.3%. Components not fully available: bicarbonate.

February 5-7, 2026 (Hospital Days 1-3): Following three days of the above regimen, reassessment revealed no significant improvement in cutaneous lesions, which continued to show a progressive trend.

February 8, 2026 (Hospital Day 4): Given the suboptimal response to initial corticosteroid therapy, the methylprednisolone dose was escalated to 60mg twice daily.

February 9, 2026 (Hospital Day 5): To further enhance supportive care, intravenous immunoglobulin (IVIG) 50g once daily and human albumin 10g once daily were added to the treatment regimen.

February 8-12, 2026 (Hospital Days 4-8): Despite four consecutive days of high-dose corticosteroid therapy (60mg BID) and the addition of IVIG support from Day 5, the cutaneous lesions remained inadequately controlled, with the clinical condition persisting as critical.

February 12, 2026 (Hospital Day 8): In view of the poor response to high-dose corticosteroids and IVIG supportive therapy, and following thorough discussion with the patient’s family regarding potential risks and benefits, off-label use of the TNF-α inhibitor adalimumab was administered as rescue therapy. Adalimumab 80mg was administered subcutaneously on this day.

February 13, 2026 (Hospital Day 9): On the day following adalimumab administration, the patient and her family requested transfer to a local dermatology specialty hospital for continued management due to personal reasons. The patient’s clinical course and treatment interventions during hospitalization are summarized in **Table 2**.

**Table 2:** Clinical course and treatment interventions.

Date	Hospital day	Clinical status	Treatment interventions
Feb 5, 2026	Day 1	Severe oral ulcers, diffuse erythema, bullae formation (BSA >30%)	Discontinued targeted therapy; Methylprednisolone 40mg QD; Sulbactam-ampicillin + Vancomycin; Supportive care
Feb 5-7	Days 1-3	No significant improvement; progressive skin involvement	Continued above regimen
Feb 8	Day 4	Persistent disease activity	Methylprednisolone escalated to 60mg BID
Feb 9	Day 5	Inadequate response to corticosteroids	Added IVIG 50g QD + Albumin 10g QD
Feb 12	Day 8	Severe disease, refractory to therapy	Added Adalimumab 80mg SC (single dose)
Feb 13	Day 9	Stable for transfer	Transferred to specialty hospital
Mid-Mar,2026	After 4 weeks of continued therapy	Markedly improved skin lesions, mucosal healing	Adalimumab+tapering corticosteroids;discharged

BSA, body surface area; QD, once daily; BID, twice daily; IVIG, intravenous immunoglobulin; SC, subcutaneous.

### Follow-up and outcome

Telephone-based post-referral follow-up was conducted. According to the patient and family, at the receiving institution, she continued adalimumab (40mg subcutaneously every 2 weeks, total 2 doses) plus a gradually tapered glucocorticoid regimen. After four weeks (until mid-March 2026), significant improvement in skin lesions was reported, including epithelial regeneration, mucosal healing, and resolution of erythema. Subsequently, she was discharged. However, the detailed treatment plan and tapering schedule from the receiving institution were unavailable, and discharge photographs were not obtainable for independent verification. Therefore, the observed improvement cannot be definitively attributed to adalimumab.

### Causality assessment

The Naranjo adverse drug reaction probability scale produced a score of 6, indicating a “probable” causal relationship between the combination therapy and the suspected SJS/TEN-like reaction. On the ALDEN scale, aumolertinib and vebreltinib scored +4 and +3, respectively, categorizing them within the “possible” to “probable” range. However, because aumolertinib and vebreltinib were initiated and withdrawn simultaneously, the specific causal contribution of each drug cannot be precisely delineated. This uncertainty should be considered when interpreting the temporal association and making future treatment decisions.

## Discussion

The value of this case lies in reporting a suspected SJS/TEN-like reaction associated with aumolertinib plus vebreltinib and in highlighting practical issues in early recognition and management. However, the diagnosis was clinical because no skin biopsy was performed, and the causal contribution of each drug could not be distinguished. The prognosis of SJS/TEN depends heavily on early intervention. Several points are noteworthy. First, the latency period—symptoms appeared on day 15 after drug initiation—aligns with the typical 4–28 day window for SJS/TEN (6). Second, mucosal involvement, especially oral ulcers, holds significant diagnostic value; approximately 80% of SJS/TEN patients have at least two mucosal sites involved (7). Third, the rash showed dangerous features, including rapid coalescence and a positive Nikolsky sign (8). When a patient presents with mucosal erosions and a rapidly progressive rash, the suspected drugs should be stopped immediately, the body surface area involvement assessed, and specialized care arranged.

Differential diagnoses that should be considered include the following. Erythema multiforme major, generalized bullous fixed drug eruption, AGEP, DRESS, and other drug exanthems may mimic SJS/TEN. In this case, the extensive epidermal detachment, mucosal erosions, positive Nikolsky sign, and rapid progression supported the clinical diagnosis of SJS/TEN-like reaction. However, skin biopsy is required for histopathological confirmation.

From a mechanistic perspective, the basis for EGFR-TKI-induced skin toxicity lies in the high expression of EGFR in keratinocytes of the epidermal basal layer, which is essential for maintaining skin barrier integrity. Inhibition of EGFR signaling leads to suppressed keratinocyte proliferation, abnormal differentiation, and release of inflammatory cytokines—the pathophysiological basis for acneiform rash (occurring in 60%-80% of cases) (9). Concurrent administration of a MET inhibitor may facilitate crosstalk between MET and EGFR pathways, leading to superimposed MAPK signaling inhibition that exacerbates keratinocyte apoptosis, the accumulation of toxic metabolites, and the activation of CD8+ T cells within the local immune microenvironment (10). TNF-α plays a central role in the pathogenesis of SJS/TEN; studies have shown significantly elevated TNF-α levels in blister fluid and serum of SJS/TEN patients, and TNF-α contributes crucially to epidermal detachment by activating the Fas-FasL pathway and directly inducing apoptosis (11). In addition, host genetic factors should not be overlooked. HLA alleles (e.g., HLA-B*15:02, HLA-B*58:01) are strongly associated with specific drug-induced SJS/TEN, with higher carrier rates in Asian populations (12). This suggests that routine screening for relevant HLA alleles should be explored for high-risk targeted combination therapies in the future.

Treatment decisions in this case must be interpreted cautiously. Immediate discontinuation of all suspected drugs is the only unequivocally effective measure, and this was achieved. The patient received methylprednisolone 40mg once daily (Days 1-3), escalated to 60mg twice daily (Days 4-8), a moderate-to-high dose (approximately 1-2mg/kg/day) but below the pulse dose used in some guidelines. Evidence for systemic corticosteroids, IVIG, and TNF-α inhibitors in SJS/TEN is limited and conflicting; treatment is often guided by clinical judgment, local protocols, and multidisciplinary discussion. No definitive recommendation can be made based on this single case. IVIG (50g/day) was started on Day 5, relatively late; whether earlier IVIG would have altered the course is unknown.

The most notable aspect is the use of adalimumab. A retrospective study by Liu et al. (2025) of 53 SJS/TEN patients suggested that adalimumab plus corticosteroids may reduce hospitalization duration, re-epithelialization time, and exposure to high-dose steroids compared with conventional treatment (13). In our patient, adalimumab was administered on Day 8 following a poor response to corticosteroids plus IVIG. After transfer, she reportedly continued adalimumab (40mg every two weeks) with tapering corticosteroids and improved. This outcome is consistent with, but does not establish, a potential benefit of adalimumab. Because the patient was transferred the next day and follow-up relied mainly on telephone reports, without complete external records or photographs, the effectiveness of adalimumab cannot be established from this case. If no response or continued progression occurs after 48–72 hours of corticosteroids plus IVIG, adding a TNF-α inhibitor may be considered after a multidisciplinary discussion; however, this should not be regarded as an established recommendation. Successful management of suspected SJS/TEN requires multidisciplinary collaboration, including early transfer to an ICU or burn unit, wound care based on burn principles, and close monitoring for infection.

## Limitations

This case presents several limitations. First, no skin biopsy was performed, so the diagnosis remains presumptive rather than histopathologically confirmed. Second, detailed treatment protocols and objective documentation from the outside hospital were unavailable, and follow-up was primarily based on telephone reports. Third, HLA genotyping was not performed. Fourth, aumolertinib and vebreltinib were started and stopped simultaneously, so the causal contribution of each drug could not be differentiated. Fifth, SCORTEN components were partially unavailable. Finally, as a single case report, these findings cannot be generalized.

## Conclusion

In summary, the combination of aumolertinib and vebreltinib may be associated with suspected SJS/TEN-like reactions—rare but potentially fatal—and clinicians should be aware of this possibility. When mucosal involvement and rapidly progressive rash appear, suspected drugs should be discontinued immediately and arrangements for emergency specialized referral made. Corticosteroids and IVIG are commonly used in clinical practice, but evidence remains limited and conflicting; no definitive recommendation can be made based on this single case. In patients with inadequate response after 48–72 hours of corticosteroid and IVIG therapy, early addition of a TNF-α inhibitor such as adalimumab may be considered after multidisciplinary discussion, but its efficacy cannot be established from this case and requires validation in larger prospective studies. Multidisciplinary collaboration, including transfer to an ICU or burn unit, specialized wound care, and infection control, should be promptly initiated. Future efforts should implement active surveillance programs for cutaneous toxicity from targeted combination therapies and explore HLA screening in high-risk populations.

## Data Availability

The raw data supporting the conclusions of this article will be made available by the authors, without undue reservation.
